# Multimodal Imaging Analysis in a Case with Congenital Fovea-Involving Retinal Macrovessel and Excellent Visual Acuity

**DOI:** 10.1155/2017/4057615

**Published:** 2017-10-19

**Authors:** Vishal Shah, M. Ashwin Reddy, Vasilios P. Papastefanou

**Affiliations:** ^1^Ophthalmology Department, The Royal London Hospital, Whitechapel, London E1 1BB, UK; ^2^Department of Paediatrics, Moorfields Eye Hospital Foundation Trust, London EC1V 2PD, UK

## Abstract

**Purpose:**

Congenital retinal macrovessels (CRM) represent rare aberrant vasculature of the retinal vessels that can supply or drain the macula. In this report, the optical coherence tomography angiography features of a congenital retinal macrovessel are discussed.

**Methods:**

The history and examination findings are presented alongside swept-source OCT angiography with corresponding B scan and en face OCT imaging.

**Patients:**

The case is a 12-year-old female patient with excellent best-corrected visual acuity in both eyes.

**Results:**

Swept-source OCT angiography demonstrated considerable loss of the foveal avascular zone at the levels of the superficial and deep capillary plexus.

**Discussion:**

In this case there was no detrimental effect on vision despite anatomical loss of the foveal avascular zone.

## 1. Introduction

Congenital retinal macrovessel was first described by Mauthner as “an aberrant retinal vessel” in 1869 [1]. Aberrant retinal vessels were noted to be rare and small in size with little or no branching and passing only to the macular region. Most were purported to arise directly from the short ciliary vessels, with occasional cases noted to communicate directly with the choroidal circulation. They were also noted to arise at both the nasal and temporal disc margins [2]. Aberrant macrovessels crossing the fovea can cause visual impairment but this is not always the case [3]. The pathophysiology is unknown; however one theory proposes a role for an intrauterine hypoxic stimulus that may then permit vascular proliferation reaching the center of the foveola [4]. In this report we describe OCT-A features in a case with congenital retinal macrovessel (CRM).

## 2. Case Presentation

**Figure 1:**
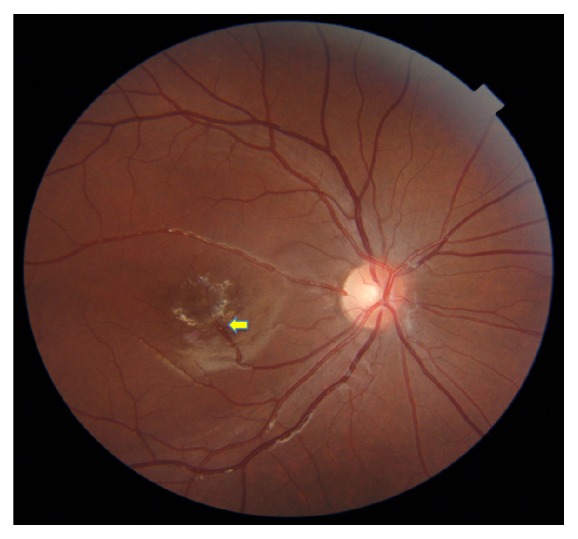
Right fundus photograph of a 12-year-old patient with a congenital retinal macrovessel (arrow) extending towards the fovea in a superotemporal course. The foveal reflex appears disturbed by the macrovessel.

A 12-year-old girl presented to the department having been referred from her opticians with an incidental finding of aberrant vasculature in the right fundus. She had no history of trauma or ocular surgery and no family history of ophthalmic disease. On examination the vision was 20/66 unaided correcting to 20/20 with glasses in the right eye and 20/16 with glasses in the left eye. Near vision was N5 bilaterally. Refraction performed at referral revealed that she was moderately myopic bilaterally (−3.50 diopters). Anterior segment examination of both eyes and intraocular pressures were normal (11 and 16 mmHg in the right and left eyes, resp.). Examination of the right fundus revealed a healthy optic disc, with an aberrant retinal macrovessel (Figure 1) arising from the inferotemporal major vascular arcade and extending towards the fovea. There were no exudates or haemorrhages and no epiretinal membrane. The appearances of the remaining retinal vessels and peripheral fundus were unremarkable. The left fundus was completely normal.

**Figure 2:**
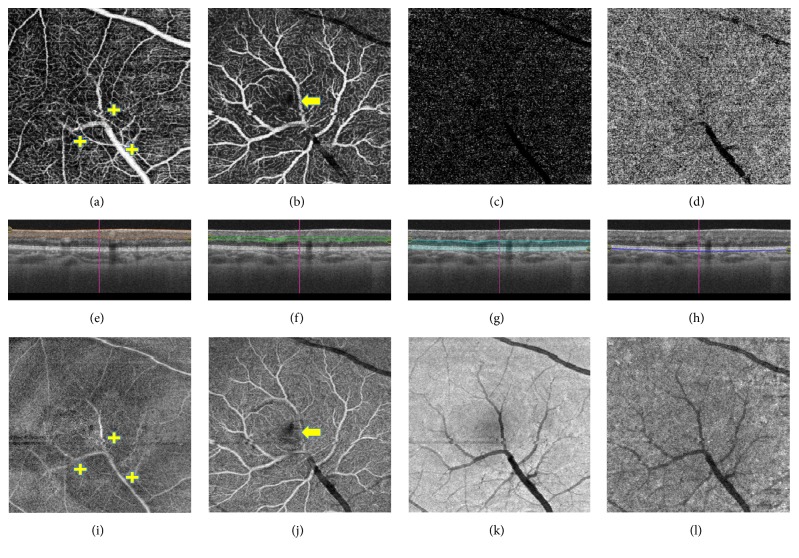
Swept-source OCT angiography (a, b, c, and d) with corresponding segmentation B scan (e, f, g, and h) and en face OCT imaging (i, j, k, and l) of the right eye of a 12-year-old patient with congenital retinal macrovessel (a, e, and i). At the level of the superficial capillary plexus the principal stem of the macrovessel and two main bifurcations are noted (+). Microvascular interconnections obliterate the foveal avascular zone (a). Corresponding SS-en face OCT does not indicate any leakage from the macrovessels (b, f, and j). At the level of the deep capillary plexus there are multiple branches emanating from the principal stem and the bifurcations directed at the level of the outer plexiform layer. The latter extend and bifurcate further horizontally prior to fusing with the capillaries of the deep vascular plexus. The remaining foveal avascular zone is also depicted (arrow), considerably reduced in extent. En face OCT also clearly demonstrates the aberrant vasculature and no evidence of leakage at this level. Scans at the level of outer retina (c, g, and k) and choriocapillaris (d, h, and l) demonstrate projection artefacts of major vessels without evidence of coexisting pathology.

**Figure 3:**
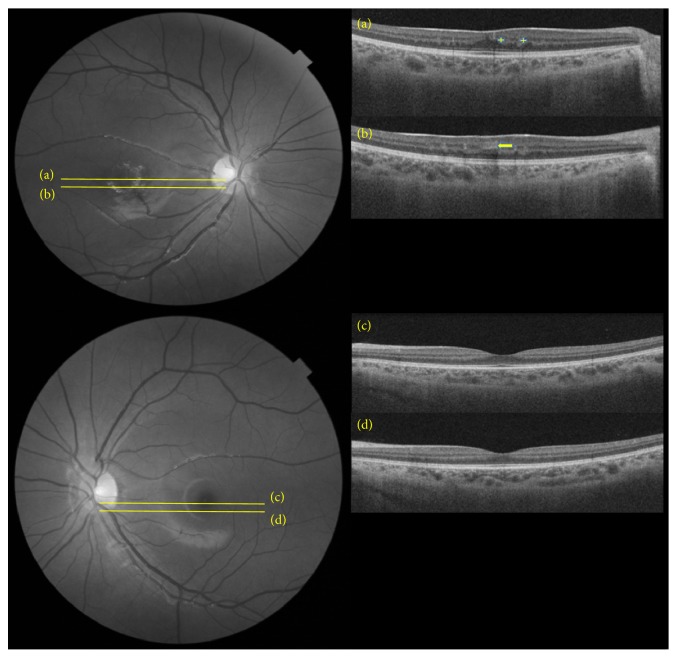
Swept-source OCT at the level of the right fovea (a) and inferior to the fovea (b) in a 12-year-old patient with congenital retinal macrovessels. The stem of the vessel is depicted at the level of the inner nuclear and plexiform layers (arrow) and its branches at the level of the outer nuclear layer (+). The foveal anatomy is largely preserved, though the foveal depression is reduced. There is no evidence of disturbance of the RPE-choriocapillaris complex also confirmed by OCT-A in the previous image. Corresponding swept-source OCT scans at the level of the left fovea (c) and inferior to the fovea (d) are included for comparison.

Swept-source optical coherence tomography angiography (SS-OCT-A) imaging of the right fundus (Triton, Topcon) (Figure 2) demonstrated the aberrant vessel encroaching into the foveal avascular zone (FAZ) but not passing through the center of the fovea. The anatomy of the neurosensory retina appeared preserved, despite the elevation of the foveal depression. No evidence of retinal layer loss, cysts, leakage, or exudation was noted. There was no evidence of disturbance in the vitreoretinal interface and no disturbance of the RPE-choriocapillaris complex (Figure 3).

Swept-source OCT angiography demonstrated considerable loss of the foveal avascular zone at the levels of the superficial and deep capillary plexus (a). Outer retinal and choriocapillaris SS-OCT-A did not yield any abnormal findings. Corresponding en face SS-OCT again confirmed no leakage from the macrovessels (b, f, and j).

## 3. Discussion

Archer et al. [5] classified congenital vessels into three categories based on the caliber of communicating vessels, presence of capillary plexus bridging the vessels, and the grade of visual impairment. Group 1 is defined as anomalous arteriovenous communications being localized to a single sector of the retina, most frequently the macula. Group 2 demonstrates direct arteriovenous communications that are larger in size than the vessels in Group 1 but in the absence of interposition of arteriolar or capillary elements. In Group 3 the arteriovenous vessels are large and entangled and give rise to retinal complications and consequent severe visual impairment. Chawla and colleagues recently reported a similar case of CRM [6]. This case was associated with poor BCVA in the affected eye since childhood and they attributed this to the distorted FAZ. This effect of aberrant retinal vasculature on vision was also emphasized in a previously reported case of a white male with two macular arteriovenous anastomoses and a small venous tributary crossing the fovea. The authors associated reduced visual acuity with the loss of the normal architecture of the fovea [7]. Goel et al. recently reported a case of reduced visual acuity due to vitreous haemorrhage secondary to congenital retinal macrovessel, highlighting the potential complications of such aberrant retinal vasculature [8]. In this case, there was no detrimental effect on vision, despite anatomical loss of the foveal avascular zone. Interestingly this disturbance of the vascular status of the macula did not considerably reflect in the anatomy of the neurosensory retina.
